# Double Quantum Image Encryption Based on Arnold Transform and Qubit Random Rotation

**DOI:** 10.3390/e20110867

**Published:** 2018-11-10

**Authors:** Xingbin Liu, Di Xiao, Cong Liu

**Affiliations:** 1 College of Computer Science, Chongqing University, Chongqing 400044, China; 2 Southwest Technology and Engineering Research Institute, Chongqing 40039, China

**Keywords:** information security, Arnold transform, quantum image encryption, quantum Fourier transform, quantum image representation

## Abstract

Quantum image encryption offers major advantages over its classical counterpart in terms of key space, computational complexity, and so on. A novel double quantum image encryption approach based on quantum Arnold transform (QAT) and qubit random rotation is proposed in this paper, in which QAT is used to scramble pixel positions and the gray information is changed by utilizing random qubit rotation. Actually, the independent random qubit rotation operates once, respectively, in spatial and frequency domains with the help of quantum Fourier transform (QFT). The encryption process accomplishes pixel confusion and diffusion, and finally the noise-like cipher image is obtained. Numerical simulation and theoretical analysis verify that the method is valid and it shows superior performance in security and computational complexity.

## 1. Introduction

Quantum computation has shown great potential for improving information processing speed and enhancing communication security [1,2,3]. The quantum image encryption technology exploits quantum mechanics principles, such as parallel and entanglement, to further protect the security of information transmission and decrease computational resource [4,5,6,7].

Due to the promising prospect of quantum image encryption, various kinds of algorithms are gradually proposed [8,9,10,11,12,13]. Among the existing algorithms, most of them are designed in spatial domain. For example, Zhou proposed a quantum image encryption algorithm using three geometric transformations, including image translation, image mirror transformation, and image sub-block swapping, which changes pixel position to some extent [14]. However, this method relies solely on geometric transformation, which leads to the increase of correlation of adjacent pixels and therefore the encryption performance is seriously affected. Except using geometric transformation to scramble pixel positions, Song proposed a novel quantum image encryption method by introducing additional color transformations, which realized pixel values diffusion and obtained better encryption results [15]. Moreover, Zhou introduced hyper-chaotic system to encrypt quantum image through XOR operation implemented with control-NOT gate [16]. Li proposed a simple color image encryption method by using 24 qubits to represent color information and employing controlled rotation gates to transform the basic state into balanced superposition state, which makes the encrypted image like a uniform white noise [17].

The quantum version of classical frequency transform tools, such as quantum Fourier transform (QFT) [18], quantum wavelet transform (QWT) [19], quantum discrete cosine transform (QDCT) [20], and promote the development of quantum image processing algorithm in frequency domain [21,22]. These quantum transforms mentioned above have lower computational complexity than their classical counterpart. Utilizing these quantum transform tools, some efficient quantum image encryption methods are investigated. Yang proposed novel quantum image encryption algorithms based on double random phase encoding framework [23], where the QFT substitutes the Fourier transform and the encryption performance of which surpasses its classical counterparts in terms of statistical analyses, robustness, and computational complexity. After that, Yang extended the quantum double random phase encoding scheme to encrypt color image [24], which introduces color image encryption into quantum scenarios in frequency domain. Recently, Li proposed a quantum encryption and compression scheme based on QDCT and a five-dimensional hyper-chaotic system [25].

Whether the quantum image encryption algorithms devised in spatial domain or frequency domain, image scrambling operation plays an important role. Jiang investigated quantum Arnold transform and Fibonacci transform method, where the quantum circuits are given and computational complexity is analyzed [26,27]. The chaos theory is also widely used in image encryption schemes [28,29,30,31,32]. Diaconu proposed a chaos based image encryption scheme by employing the circular inter-intra permutation strategy [33]. Stoyanov presented a Chebyshev polynomial based image encryption scheme, which shows the advantage in terms of key space [34]. Parvees utilized logistic map and key image to efficiently encrypt large size image [35]. Soon afterwards, Zhou suggested the generalized Arnold transform with feature of chaotic mapping [36,37]. In addition, a generalized quantum affine transform is proposed to scramble images, which can encode pixel positions effectively [38]. Quantum Hilbert scrambling method is also introduced and achieves good permutation effect [39]. These scrambling methods adopt position space scrambling strategies, which do not change color space. To overcome this defect, Zhou proposed a bit-plane scrambling method that is based on Gray-code [40], which simultaneously changes the pixel positions and pixel values, and it even can be used directly to encrypt images. By combining the bit-plane scrambling method and the Hilbert scrambling method, Naseri proposed a quantum gray-scale image encoding scheme, where a randomly generated binary key is used to select encoding scheme [41].

The existing quantum image encryption algorithms mainly focus on single gray or color image, while the research on double quantum image encryption or multiple quantum image encryption is still scarce. In view of this, a double quantum image encryption algorithm that is based on quantum Arnold transform (QAT) and qubit random rotation is proposed. Firstly, the two images to be encrypted are represented through a flexible quantum image representation model called flexible representation for quantum images (FRQI). Next, the two quantum states are scrambled using QAT with different parameters, and one of the scrambled quantum images is encoded into amplitude part and another is encoded into phase part. Then the independent random qubit rotation operates once, respectively, in spatial and frequency domains with the help of quantum Fourier transform (QFT) to accomplish pixel confusion and diffusion. The noise-like cipher image can be finally obtained by performing inverse QFT. The original images can be exactly recovered without cross-talk. The quantum parallel computation speeds up the process of double image encryption and decryption. Numerical simulation results and theoretical analyses demonstrate that the proposed algorithm is effective and the computational complexity is decreased.

## 2. Preliminary Knowledge 

### 2.1. FRQI Representation Model

The first step of quantum image processing is to design a suitable representation model, which can be run on quantum computers for compiling digital image. Nowadays, several efficient representation models are proposed [3]. The FRQI representation model [42] is widely used, as it is similar with pixel representation in classical computer and accord with human perception of vision. 

The FRQI representation model stores gray and geometric information using a normalized quantum state. For a gray image M of size $2^{n}\times 2^{n}$, the representation can be expressed, as follows,

(1) $$|M\left(\theta \right)\rangle =\frac{1}{2^{n}}\sum_{i=0}^{2^{2n}-1}|c_{i}\rangle ⊗|i\rangle ,\;i=0,1,2,\cdots ,2^{2n}-1$$

(2) $$|c_{i}\rangle =\cos\theta_{i}|0\rangle +\sin\theta_{i}|1\rangle ,\;\theta_{i}\in \left[0,\pi /2\right]$$

where $\theta =\left(\theta_{1},\theta_{2},\cdots ,\theta_{2^{2n}-1}\right)$ is the vector used to encode phase information of gray values and $|i\rangle =|y\rangle |x\rangle =|y_{n-1}y_{n-2}\cdots y_{0}\rangle |x_{n-1}x_{n-2}\cdots x_{0}\rangle$ is used to encode corresponding pixel positions of θ. The symbol ⊗ denotes tensor product. There are only 2n+1 qubits required when encoding image and the computational complexity of preparation process is $O\left(2^{4n}\right)$.

### 2.2. Quantum Arnold Transform (QAT)

The Arnold transform, used as method for image pixel scrambling, is built on the research of ergodic theory and it was extended to quantum image processing in 2014 by Jiang et al. [26]. QAT aims to transform the image into a confused form through changing the coordinates of pixels. 

Suppose that the image of size $2^{n}\times 2^{n}$ to be scrambled is denoted as I(x,y), where (x,y) represent pixel positions. A two-dimensional Arnold transform is described, as follows,

(3) $$\left(\begin{array}{l} x^{\prime }\\ y^{\prime } \end{array}\right)=\left(\begin{array}{cc} 1 & 1\\ 1 & 2 \end{array}\right)\left(\begin{array}{l} x\\ y \end{array}\right)\left(\mathrm{mod}\;2^{n}\right),\;x,y=0,1,\cdots ,2^{n}$$

i.e.,

(4) $$\left\{ \begin{array}{l} x^{\prime }=\left(x+y\right)\mathrm{mod}\;2^{n}\\ y^{\prime }=\left(x+2y\right)\mathrm{mod}\;2^{n} \end{array}\right.$$

The output $\left(x^{\prime },y^{\prime }\right)$ is the scrambled position information and the inverse transform can be deduced as follows,

(5) $$\left(\begin{array}{l} x\\ y \end{array}\right)={\left(\begin{array}{cc} 1 & 1\\ 1 & 2 \end{array}\right)}^{-1}\left(\begin{array}{l} x^{\prime }\\ y^{\prime } \end{array}\right)\left(\mathrm{mod}\;2^{n}\right)=\left(\begin{array}{cc} 2 & -1\\ -1 & 1 \end{array}\right)\left(\begin{array}{l} x^{\prime }\\ y^{\prime } \end{array}\right)\left(\mathrm{mod}\;2^{n}\right)$$

**Figure 1:**
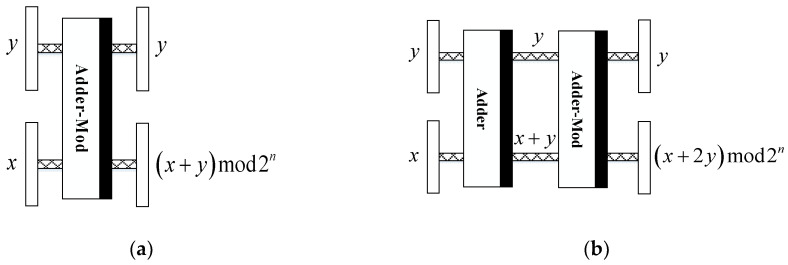
The scrambling circuits for (**a**) $x^{\prime }$ and (**b**) $y^{\prime }$.

The scrambling period of Arnold transform is associated with the size of image, and some determined values for particular cases are given in Table 1. Although the period of Arnold transform cannot be accurately computed, it can be seen that the period grows with the increase of image size. The quantum circuits shown in Figure 1 are used to accomplish the QAT scrambling. The detailed information of quantum adder module and ${\text{adder-mod}2}^{n}$ module is presented in [27].

## 3. Proposed Double Quantum Image Encryption Scheme

In this section, the proposed double quantum image encryption scheme based on QAT and qubit random rotation is illustrated in detail. Let the two images to be encrypted be respectively denoted as $I_{1}$ and $I_{2}$. According to the FRQI representation model, the original images can be represented as follows,

(6) $$\begin{array}{l} |I_{1}\left(\theta \right)\rangle =\frac{1}{2^{n}}\sum_{y=0}^{2^{n}-1}\sum_{x=0}^{2^{n}-1}|\alpha_{yx}\rangle ⊗|yx\rangle \\ |I_{2}\left(\omega \right)\rangle =\frac{1}{2^{n}}\sum_{y=0}^{2^{n}-1}\sum_{x=0}^{2^{n}-1}|\beta_{yx}\rangle ⊗|yx\rangle \end{array}$$

where the gray values are represented as $|\alpha_{yx}\rangle =\cos\theta_{yx}|0\rangle +\sin\theta_{yx}|1\rangle$, $|\beta_{yx}\rangle =\cos\omega_{yx}|0\rangle +\sin\omega_{yx}|1\rangle$, and $\left\{\theta_{yx},\omega_{yx}\right\}\in \left[0,\pi /2\right]$. The whole double quantum image encryption algorithm consists the following five steps.

***Step 1.*** Scramble the two original images $|I_{1}\rangle$ and $|I_{2}\rangle$ in spatial domain using QAT to get $|I_{1}^{\prime }\rangle$ and $|I_{2}^{\prime }\rangle$. The parameters of QAT corresponding to $|I_{1}\rangle$ and $|I_{2}\rangle$ are respectively denoted as $p_{1}$ and $p_{2}$, which represent the iteration times of scrambling.

(7) $$\begin{array}{ll} |I_{1}^{\prime }\rangle & ={\mathrm{QAT}}_{p_{1}}\left(|I_{1}\rangle \right)=\frac{1}{2^{n}}\sum_{y=0}^{2^{n}-1}\sum_{x=0}^{2^{n}-1}|\alpha_{yx}\rangle ⊗{\mathrm{QAT}}_{p_{1}}\left(|yx\rangle \right)\\ & =\frac{1}{2^{n}}\sum_{y=0}^{2^{n}-1}\sum_{x=0}^{2^{n}-1}|\alpha_{yx}\rangle ⊗\left(|y^{\prime }x^{\prime }\rangle \right) \end{array}$$

(8) $$\begin{array}{ll} |I_{2}^{\prime }\rangle & ={\mathrm{QAT}}_{p_{2}}\left(|I_{2}\rangle \right)=\frac{1}{2^{n}}\sum_{y=0}^{2^{n}-1}\sum_{x=0}^{2^{n}-1}|\beta_{yx}\rangle ⊗{\mathrm{QAT}}_{p_{2}}\left(|yx\rangle \right)\\ & =\frac{1}{2^{n}}\sum_{y=0}^{2^{n}-1}\sum_{x=0}^{2^{n}-1}|\beta_{yx}\rangle ⊗\left(|y^{\prime }x^{\prime }\rangle \right) \end{array}$$

where $\mathrm{QAT}\left(|yx\rangle \right)=|\left(x+2y\right)\mathrm{mod}\;2^{n}\rangle |\left(x+y\right)\mathrm{mod}\;2^{n}\rangle$. There are a total of $p_{1}$ times scrambling operations for quantum image $|I_{1}\rangle$ and $p_{2}$ times for $|I_{2}\rangle$.

***Step 2***. Encode the scrambled image $|I_{2}^{\prime }\rangle$ into a phase function and the scrambled image $|I_{1}^{\prime }\rangle$ is regarded as amplitude. Then, a new complex image $|I^{\prime }\rangle$ involving all the information of the two original images can be expressed, as follows,

(9) $$\begin{array}{ll} |I^{\prime }\rangle & =|I_{1}^{\prime }\rangle \exp\left(i\pi |I_{2}^{\prime }\rangle \right)\\ & =\frac{1}{2^{n}}\sum_{y=0}^{2^{n}-1}\sum_{x=0}^{2^{n}-1}|\alpha_{yx}\rangle \exp\left(i\pi |\beta_{yx}\rangle \right)⊗|y^{\prime }x^{\prime }\rangle \end{array}$$

***Step 3.*** Perform qubit random rotation on quantum image $|I^{\prime }\rangle$ to transform its each gray value angle into a new angle. The rotation matrix $R_{yx}\left(\phi_{yx}\right)$ defined as follows is used to change angles in spatial domain,

(10) $$R_{yx}\left(\phi_{yx}\right)=\left[\begin{array}{cc} \cos\phi_{yx} & -\sin\phi_{yx}\\ \sin\phi_{yx} & \cos\phi_{yx} \end{array}\right]$$

where $\phi_{yx}$ is uniformly distributed in the interval [0,2π]. The controlled rotation matrix $CR_{YX}\left(\phi_{YX}\right)$ defined as follows is used to change angles in position (Y,X),

(11) $$CR_{YX}\left(\phi_{YX}\right)=I⊗\sum_{y=0}^{2^{n}-1}\sum_{\begin{array}{l} x=0\\ yx\neq YX \end{array}}^{2^{n}-1}|yx\rangle \langle yx|+R_{YX}\left(\phi_{YX}\right)⊗|YX\rangle \langle YX|$$

The controlled rotation matrix $CR_{YX}\left(\phi_{YX}\right)$ is a unitary matrix because of $CR_{YX}CR_{YX}^{†}=I{⊗}^{2n+1}$. The $CR_{YX}^{†}$ represents the Hermitian conjugate of $CR_{YX}$ and the symbol I denotes unit matrix. 

In order to complete the rotation of all positions, the products of $2^{2n}$ controlled rotation matrices are applied on quantum image $|I^{\prime }\rangle$ and obtain $|E_{1}\rangle$.

(12) $$\begin{array}{ll} CR\left(|I^{\prime }\rangle \right) & =\prod_{Y=0}^{2^{n}-1}\prod_{X=0}^{2^{n}-1}CR_{YX}\left(|I^{\prime }\rangle \right)\\ & =\frac{1}{2^{n}}\sum_{y=0}^{2^{n}-1}\sum_{x=0}^{2^{n}-1}R_{yx}\left(|\alpha_{yx}\rangle \exp\left(\text{i}\pi |\beta_{yx}\rangle \right)\right)⊗|y^{\prime }x^{\prime }\rangle \\ & =\frac{1}{2^{n}}\sum_{y=0}^{2^{n}-1}\sum_{x=0}^{2^{n}-1}|f_{yx}\rangle ⊗|y^{\prime }x^{\prime }\rangle \\ & =|E_{1}\rangle \end{array}$$

***Step 4.*** Transform the obtained $|E_{1}\rangle$ into frequency domain using QFT. The QFT is the identical transform of discrete Fourier transform, which is defined as follows,

(13) $$\mathrm{QFT}\left(|i\rangle \right)=\frac{1}{\sqrt{N}}\sum_{j=0}^{N-1}e^{2\pi ijk/N}|j\rangle$$

Similar to the rotation in spatial domain, qubit random rotation is employed in the frequency domain. Another rotation matrix $T_{yx}\left(\psi_{yx}\right)$ defined as follows is used,

(14) $$T_{yx}\left(\psi_{yx}\right)=\left[\begin{array}{cc} \cos\psi_{yx} & -\sin\psi_{yx}\\ \sin\psi_{yx} & \cos\psi_{yx} \end{array}\right]$$

where $\psi_{yx}$ is also uniformly distributed in the interval [0,2π]. The controlled rotation matrix $CT_{YX}\left(\psi_{yx}\right)$ is defined, as follows,

(15) $$CT_{YX}\left(\psi_{YX}\right)=I⊗\sum_{y=0}^{2^{n}-1}\sum_{\begin{array}{l} x=0\\ yx\neq YX \end{array}}^{2^{n}-1}|yx\rangle \langle yx|+T_{YX}\left(\psi_{YX}\right)⊗|YX\rangle \langle YX|$$

The controlled rotation matrix $CT_{YX}\left(\psi_{YX}\right)$ is also a unit matrix and $CT_{YX}CT_{YX}^{†}=I{⊗}^{2n+1}$.

The rotation of frequency domain can be accomplished using the product of $2^{2n}$ controlled rotation matrices operate on quantum image $|E_{1}\rangle$ and obtain $|E_{2}\rangle$.

(16) $$\begin{array}{l} CT\left(\text{QFT}\left(|E_{1}\rangle \right)\right)=\prod_{Y=0}^{2^{n}-1}\prod_{X=0}^{2^{n}-1}CT_{YX}\left(\text{QFT}\left(|E_{1}\rangle \right)\right)\\ \;=\frac{1}{2^{n}}\sum_{y=0}^{2^{n}-1}\sum_{x=0}^{2^{n}-1}\left(\text{QFT}\left(|\alpha_{yx}\rangle \exp\left(i\pi |\beta_{yx}\rangle \right)\right)\right)⊗|y^{\prime }x^{\prime }\rangle \\ \;=\frac{1}{2^{n}}\sum_{y=0}^{2^{n}-1}\sum_{x=0}^{2^{n}-1}T_{yx}\left(\text{QFT}\left(|f_{yx}\rangle \right)\right)⊗|y^{\prime }x^{\prime }\rangle \\ \;=|E_{2}\rangle \end{array}$$

***Step 5.*** Execute the inverse quantum Fourier transform (iQFT) and the final encrypted quantum image |E〉 is obtained.

(17) $$\begin{array}{ll} |E\rangle & =\mathrm{iQFT}\left(|E_{2}\rangle \right)\\ & =\frac{1}{2^{n}}\mathrm{iQFT}\left(\sum_{y=0}^{2^{n}-1}\sum_{x=0}^{2^{n}-1}T_{yx}\left(\mathrm{QFT}\left(R_{yx}\left(|\alpha_{yx}\rangle \exp\left(i\pi |\beta_{yx}\rangle \right)\right)\right)\right)⊗|y^{\prime }x^{\prime }\rangle \right) \end{array}$$

The decryption scheme is just the inverse of the aforementioned encryption scheme as the quantum transformations used are unitary and invertible. The keys including parameters of QAT $p_{1}$ and $p_{2}$, two rotation matrices R(ϕ) and T(ψ) are needed to correctly decrypt the cipher image. Corresponding to the encryption procedure, the decryption process can be expressed, as follows.

***Step 1.*** Perform QFT on the encrypted quantum image |E〉 and obtain $|E_{2}\rangle$,

(18) $$\mathrm{QFT}\left(|E\rangle \right)=\mathrm{QFT}\left(\mathrm{iQFT}\left(|E_{2}\rangle \right)\right)=|E_{2}\rangle$$

***Step 2.*** Execute quantum rotation on $|E_{2}\rangle$ using the key T(ψ).

(19) $$\begin{array}{ll} CT^{-1}\left(|E_{2}\rangle \right) & =\prod_{Y=0}^{2^{n}-1}\prod_{X=0}^{2^{n}-1}CT_{YX}^{†}\left(|E_{2}\rangle \right)\\ & =\prod_{Y=0}^{2^{n}-1}\prod_{X=0}^{2^{n}-1}CT_{YX}^{†}\left(\frac{1}{2^{n}}\sum_{y=0}^{2^{n}-1}\sum_{x=0}^{2^{n}-1}T_{yx}\left(\text{QFT}\left(|f_{yx}\rangle \right)\right)⊗|y^{\prime }x^{\prime }\rangle \right)\\ & =\frac{1}{2^{n}}\sum_{y=0}^{2^{n}-1}\sum_{x=0}^{2^{n}-1}T_{yx}^{-1}T_{yx}\left(\text{QFT}\left(|f_{yx}\rangle \right)\right)⊗|y^{\prime }x^{\prime }\rangle \\ & =\frac{1}{2^{n}}\sum_{y=0}^{2^{n}-1}\sum_{x=0}^{2^{n}-1}\text{QFT}\left(|f_{yx}\rangle \right)⊗|y^{\prime }x^{\prime }\rangle \\ & =\text{QFT}\left(|E_{1}\rangle \right) \end{array}$$

***Step 3***. Apply iQFT on the result that was obtained in previous step and $|E_{1}\rangle$ is attained. Then, the qubit rotation is operated on $|E_{1}\rangle$ with the key $R_{yx}\left(\phi_{yx}\right)$. This step can be expressed as follows,

(20) $$\mathrm{iQFT}\left(\mathrm{QFT}\left(|E_{1}\rangle \right)\right)=|E_{1}\rangle$$

(21) $$\begin{array}{ll} CR^{-1}\left(|E_{1}\rangle \right) & =\prod_{Y=0}^{2^{n}-1}\prod_{X=0}^{2^{n}-1}CR_{YX}^{†}\left(|E_{1}\rangle \right)\\ & =\prod_{Y=0}^{2^{n}-1}\prod_{X=0}^{2^{n}-1}CR_{YX}^{†}\left(\frac{1}{2^{n}}\sum_{y=0}^{2^{n}-1}\sum_{x=0}^{2^{n}-1}R_{yx}\left(|f_{yx}\rangle \right)⊗|y^{\prime }x^{\prime }\rangle \right)\\ & =\frac{1}{2^{n}}\sum_{y=0}^{2^{n}-1}\sum_{x=0}^{2^{n}-1}R_{yx}^{-1}R_{yx}\left(|f_{yx}\rangle \right)⊗|y^{\prime }x^{\prime }\rangle \\ & =\frac{1}{2^{n}}\sum_{y=0}^{2^{n}-1}\sum_{x=0}^{2^{n}-1}|f_{yx}\rangle ⊗|y^{\prime }x^{\prime }\rangle \\ & =|I^{\prime }\rangle \end{array}$$

***Step 4.*** Extract two quantum images $|I_{1}^{\prime }\rangle$ and $|I_{2}^{\prime }\rangle$ from $|I^{\prime }\rangle$,

(22) $$\left\{ \begin{array}{l} |I_{1}^{\prime }\rangle =\mathrm{abs}(|I^{\prime }\rangle )\\ |I_{2}^{\prime }\rangle =\mathrm{angle}(|I^{\prime }\rangle )/\pi \end{array}\right.$$

where abs(⋅) and angle(⋅) denote the extraction of amplitude and phase, respectively.

***Step 5.*** Execute inverse QAT (iQAT) on quantum images $|I_{1}^{\prime }\rangle$ and $|I_{2}^{\prime }\rangle$ with keys $p_{1}$ and $p_{2}$, thus the original images $|I_{1}\rangle$ and $|I_{2}\rangle$ are decrypted.

(23) $$\begin{array}{ll} |I_{1}\rangle & ={\mathrm{iQAT}}_{p_{1}}\left(|I_{1}^{\prime }\rangle \right)\\ & ={\mathrm{iQAT}}_{p_{1}}\left(\frac{1}{2^{n}}\sum_{y=0}^{2^{n}-1}\sum_{x=0}^{2^{n}-1}|\alpha_{yx}\rangle ⊗\left(|y^{\prime }x^{\prime }\rangle \right)\right)\\ & =\frac{1}{2^{n}}\sum_{y=0}^{2^{n}-1}\sum_{x=0}^{2^{n}-1}|\alpha_{yx}\rangle ⊗{\mathrm{iQAT}}_{p_{1}}\left(|y^{\prime }x^{\prime }\rangle \right)\\ & =\frac{1}{2^{n}}\sum_{y=0}^{2^{n}-1}\sum_{x=0}^{2^{n}-1}|\alpha_{yx}\rangle ⊗|yx\rangle \end{array}$$

(24) $$\begin{array}{ll} |I_{2}\rangle & ={\mathrm{iQAT}}_{p_{2}}\left(|I_{2}^{\prime }\rangle \right)\\ & ={\mathrm{iQAT}}_{p_{2}}\left(\frac{1}{2^{n}}\sum_{y=0}^{2^{n}-1}\sum_{x=0}^{2^{n}-1}|\beta_{yx}\rangle ⊗\left(|y^{\prime }x^{\prime }\rangle \right)\right)\\ & =\frac{1}{2^{n}}\sum_{y=0}^{2^{n}-1}\sum_{x=0}^{2^{n}-1}|\beta_{yx}\rangle ⊗{\mathrm{iQAT}}_{p_{2}}\left(|y^{\prime }x^{\prime }\rangle \right)\\ & =\frac{1}{2^{n}}\sum_{y=0}^{2^{n}-1}\sum_{x=0}^{2^{n}-1}|\beta_{yx}\rangle ⊗|yx\rangle \end{array}$$

where ${\mathrm{iQAT}}_{p_{1}}$ denotes operate $p_{1}$ times iQAT on pixel position $|y^{\prime }x^{\prime }\rangle$ and ${\mathrm{iQAT}}_{p_{2}}$ operates in a similar way. The iQAT can be expressed, as follows,

(25) $$\begin{array}{ll} \mathrm{iQAT}\left(|y^{\prime }x^{\prime }\rangle \right) & =\mathrm{iQAT}\left(|y^{\prime }\rangle |x^{\prime }\rangle \right)\\ & =|\left(-x^{\prime }+y^{\prime }\right)\mathrm{mod}2^{n}\rangle |\left(2x^{\prime }-y^{\prime }\right)\mathrm{mod}2^{n}\rangle \\ & =|y\rangle |x\rangle =|yx\rangle \end{array}$$

## 4. Numerical Simulation and Discussion

**Figure 2:**
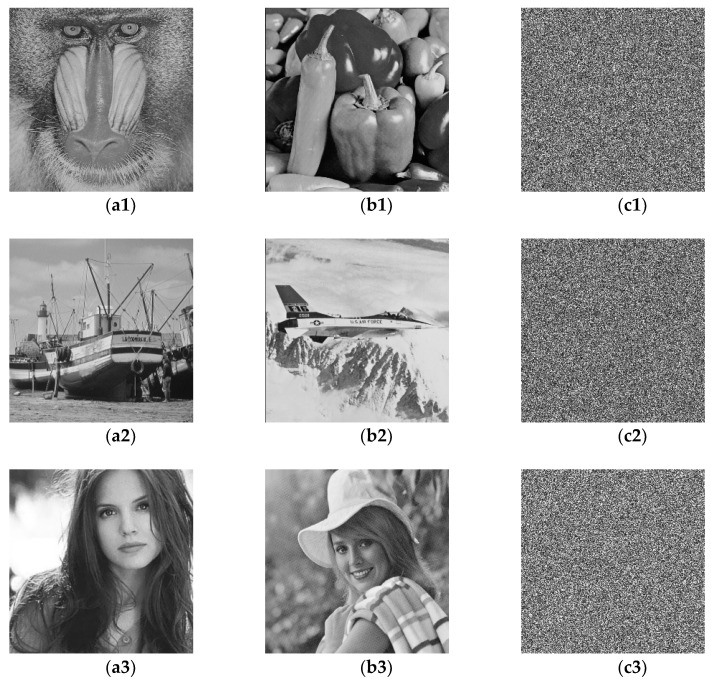
Three pairs of original images and corresponding cipher images.

Due to the lack of quantum hardware to implement the proposed double image encryption algorithm, numerical simulations are made on a classical computer with the MATLAB software (R2017a, MathWorks, Natick, MA, USA). The quantum states and quantum transformations can be simulated using complex vectors and unitary matrices. Therefore, the MATLAB is good at dealing with linear algebra is selected as the simulation tool. The size of all the original images is 256×256 and the period of QAT is 192. The image is scrambled when the parameter of QAT is not exactly equal to the multiple of period. The randomly selected iteration times of QAT can be severed as keys and they are set to $p_{1}=42$ and $p_{2}=73$ in the experiment. The rotation matrices R(ϕ) and T(ψ) are randomly generated. Three pairs of original images and corresponding cipher images are shown in Figure 2, from which can be seen that the encrypted images are noise-like and security analyses are given in the following subsections.

### 4.1. Histogram Analysis

**Figure 3:**
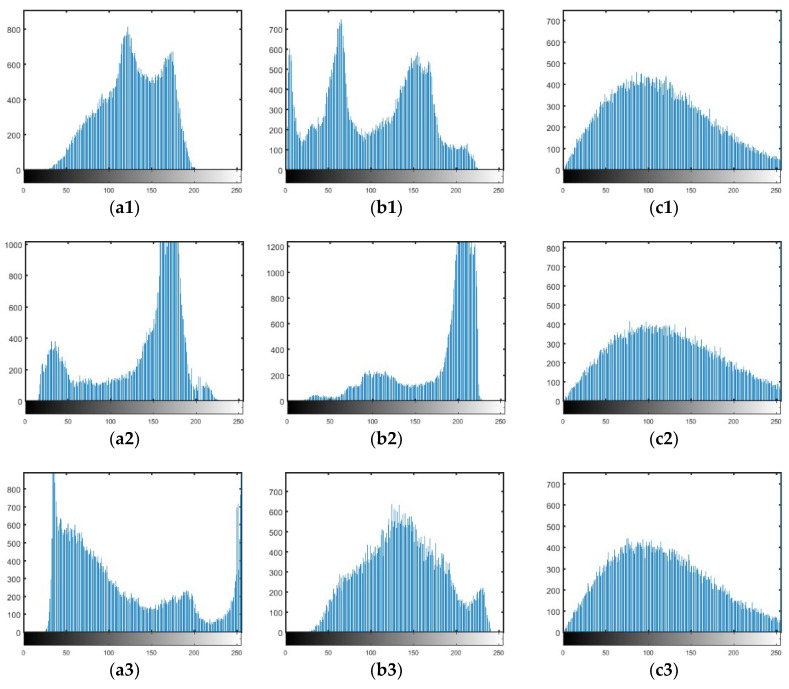
The histograms of original images and cipher images.

The gray histogram is generally used to view the pixel distribution by count the frequency of pixels in all gray level. The histograms corresponding to Figure 2 are plotted in Figure 3, from which can be seen that the histogram of each original image is different from each other, but the histograms of all the cipher images are similar. In addition, the histograms of the cipher images are smoother. Therefore, there is no clue for eavesdroppers performing statistical attack or differential attack on the encrypted images and any useful information cannot be obtained.

### 4.2. Correlation Analysis

The adjacent pixels in natural images are highly correlated while such correlation should be break for an ideal image encryption scheme. To verify the confusion and diffusion effect of the proposed double quantum image encryption algorithm, the correlation coefficients (*CC*) in horizontal, vertical, and diagonal directions are computed. Moreover, the correlation distributions are plotted.

The *CC* value of adjacent pixels is defined, as follows,

(26) $$CC=\frac{\sum_{l=1}^{N}\left(u_{l}-\bar{u}\right)\left(v_{l}-\bar{v}\right)}{\sqrt{\sum_{l=1}^{N}{\left(u_{l}-\bar{u}\right)}^{2}\sum_{l=1}^{N}{\left(v_{l}-\bar{v}\right)}^{2}}}$$

where u and v represent two adjacent pixel values. The $\bar{u}$ and $\bar{v}$ denote mean value, i.e., $\bar{u}=\sum_{l=1}^{N}u_{l}/N$ and $\bar{v}=\sum_{l=1}^{N}v_{l}/N$.

Take the images shown in Figure 2 as example to test the correlation of adjacent pixels, and the *CC* values in three directions are listed in Table 2. It can be seen that the *CC* values in the cipher image are close to 0 in three directions, which means that the correlation is greatly decreased in cipher images. 

**Figure 4:**
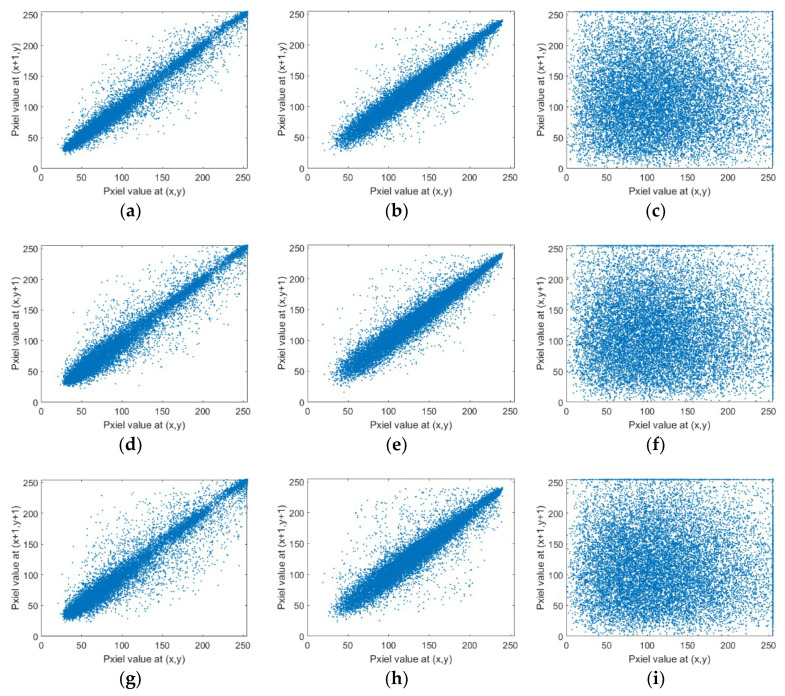
The distribution of adjacent pixels in horizontal, vertical and diagonal directions.

In addition, in order to visualize the correlation distribution of original images and the cipher image, 16,000 pairs of adjacent pixels are randomly selected from each direction. Take the images in third group as example, the distributions in horizontal, vertical, and diagonal directions are respectively shown in Figure 4a–i. The first row shows the horizontal distributions and second row shows the vertical distribution, and the diagonal direction is shown in the last row. From the distribution, figures of adjacent pixels can be seen that the proposed algorithm breaks the high correlation in original images and therefore the eavesdroppers cannot obtain information from the statistical analysis.

### 4.3. Information Entropy

The information entropy can reflect the spatial feature of gray distribution, which is defined using the probability of gray value. As the gray level of experimental images is 0–255, suppose that the probability of gray value i is P(i), then the information entropy IE can be calculated as

(27) $$IE=-\sum_{i=0}^{255}P\left(i\right)\log_{2}P\left(i\right)$$

The value of IE grows with the degree of confusion and the ideal value for encrypted image should be 8. Table 3 lists the IE values of original images and cipher images, from which can be seen that information entropy is increased. The results of information entropy coincided with the correlation analysis.

### 4.4. Noise Robustness

**Figure 5:**
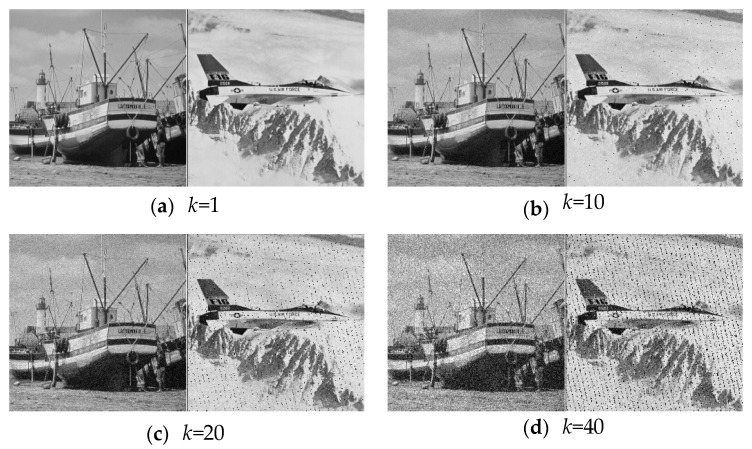

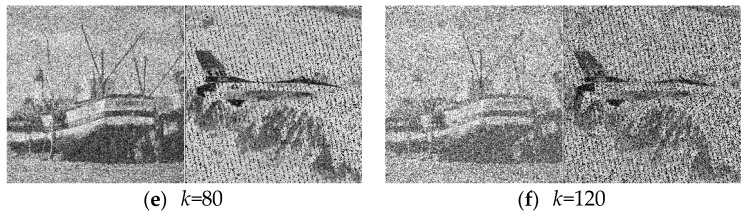
The recovered results of different noise intensity attack.

The images are usually interfered with noises during processing or transmission, which decrease the quality of decrypted images. In order to value the robustness of anti-noise of the proposed algorithm, Gussian noises with different intensity are added to the encrypted images. Take the second group of image as example, let E denote the encrypted image and $E^{\prime }$ represents the noisy encrypted image, then the noise adding process can be expressed as

(28) $$E^{\prime }=E+kG$$

where G denotes (0,1) Gussian noise and k is noise intensity. The decrypted images with different noise intensity are shown in Figure 5a–f. Although the noise intensity increases to 120, the original information can still be recognized.

**Figure 6:**
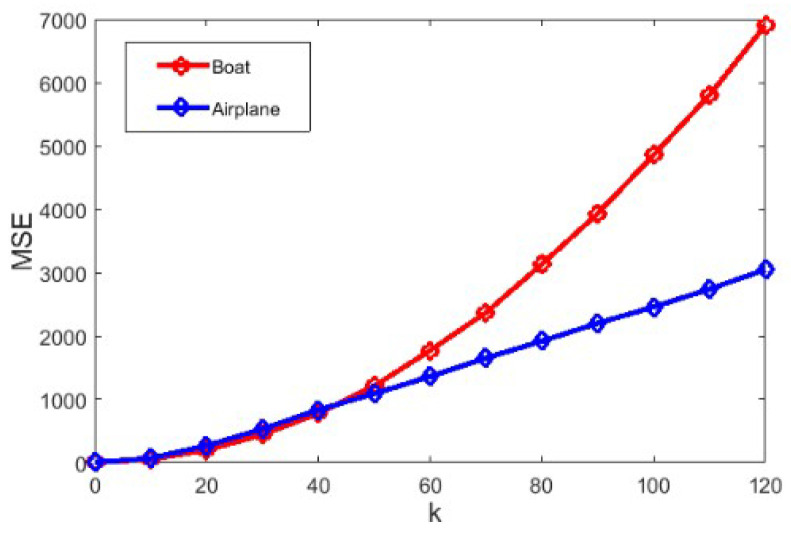
The mean square error (MSE) curves under different intensities of noise.

In addition, the mean square error (MSE) is introduced to quantitative compare the difference of between the decrypted image and the original image. The MSE is defined as

(29) $$MSE=\frac{\sum_{i=1}^{2n}\sum_{j=1}^{2n}{\left(D\left(i,j\right)-I\left(i,j\right)\right)}^{2}}{2^{n}\times 2^{n}}$$

where D(i,j) and I(i,j) denote the decrypted image and the original image, respectively. The MSE curves under different intensities of noise are plotted in Figure 6. In combination with the decrypted images that are shown in Figure 5, it can be seen that the proposed algorithm performs good in resisting noise attacks. It also can be concluded that the QAT scrambling process improves the performance of anti-noise in some degree.

### 4.5. Key Sensitivity Analysis

**Figure 7:**
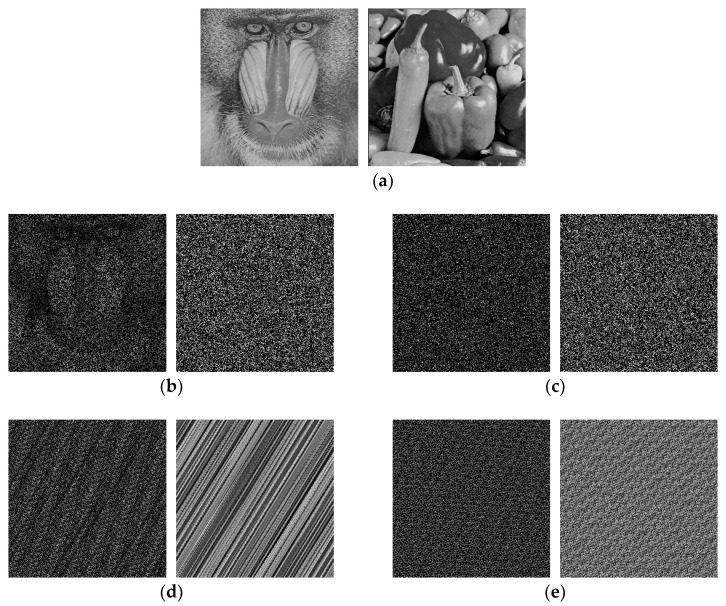
The decryption result with (**a**) correct keys, (**b**) incorrect random rotation matrix R(ϕ), (**c**) incorrect random rotation matrix T(ψ), (**d**) incorrect quantum Arnold transform (QAT) parameters, and (**e**) another pair of incorrect QAT parameters.

For a good image cryptosystem, the cipher key should be sensitive to secure it against brute-force attack. In the proposed algorithm, the keys include two independent random rotation matrices and two parameters of QAT. Take the first group of image as an example, the decrypted images with correct keys are shown in Figure 7a. Figure 7b shows the decrypted images with incorrect random rotation matrix R(ϕ), from which can be seen that the recovered images are blurry. Figure 7c shows the decrypted images with incorrect random rotation matrix T(ψ), from which can be seen that the recovered images are noise-like and any useful information cannot be obtained. The decrypted images with incorrect parameters of QAT are shown in Figure 7d,e, where the deviations of parameters are 3 and 8 respectively. Obviously, the original image can be successfully recovered when all the keys are correct.

In addition, the key space of the proposed algorithm is analyzed. To resist brute-force attack, the key space should larger than $2^{100}$ under current computation ability. As the keys used are independent, the total key space is the product of a single key space. The key space for QAT is about $2^{15}$. The key space for random rotation matrix depends on the size of image, which is larger than $2^{256\times 256}$. Therefore, the key space is large enough to ensure the security of the proposed algorithm. In addition, the key space of the proposed algorithm is compared with several state of art image encryption algorithms [26,27,32,33,34]. The comparison results that are shown in Table 4 indicate that the proposed algorithm has a larger key space. 

### 4.6. Computational Complexity Analysis

The computational complexity of the proposed algorithm and classical counterpart is analyzed in this subsection. The complexity of quantum algorithm depends on the basic logical element such as Control-NOT gate and NOT gate. The complexity of quantum adder is about 28n [25]. The adder-mod module includes five adder modules and therefore the complexity of QAT is about 140n. As the proposed algorithm uses twice QAT and the computational complexity in QAT scrambling is estimated to 280n. In addition, the quantum circuits of QFT including n(n−1)/2 basic gates and random rotation operation has a O(n) complexity. Therefore, the complexity of the proposed algorithm is $O\left(n^{2}\right)$. The computational complexity of each step and overall complexity is shown in Table 5. By contrast, if this algorithm runs on a classical computer, all the operations are performed on every pixel, then the complexity of Arnold transform and angle rotation is $2^{2n}$. Besides, the computational complexity of Fourier transform is $O\left(n2^{2n}\right)$. Therefore, the complexity of the classical algorithm is $O\left(n2^{2n}\right)$, which is more complex than the quantum one. In a conclusion, the proposed double quantum image encryption algorithm performs better than its classical counterpart in the aspect of computational complexity. 

## 5. Conclusions

In this paper, a double quantum gray image encryption algorithm that is based on QAT and quantum random rotation is proposed. The main contribution of this paper lies in encrypting double quantum gray images by combining quantum permutation and qubits angle random rotation, which further improves the encryption efficiency. The original two images can be completely retrieved without distortion and cross-talk through using correct keys. The key space of the proposed method is larger than the compared methods, which ensures the security to resist brute-force attack. Experimental results and theoretical analysis show that the proposed algorithm is robustness to resist statistical attack and noise attack. Moreover, the proposed algorithm is superior compared with its counterpart in terms of computational complexity.

There are also some disadvantages in the proposed scheme, such as the histogram of the ciphertext image, is not uniformly distributed. In addition, the color image usually presents abundant information, so color image encryption should be paid more attention. We will focus on solving disadvantages and putting forward double color image encryption schemes in our future research.

## Figures and Tables

**Table 1:** The scrambling period of Arnold transform.

Image Size	Period
16×16	12
32×32	24
64×64	48
128×128	96
256×256	192

**Table 2:** Correlation coefficients of original images and cipher images.

Correlation Coefficient	Horizontal	Vertical	Diagonal
Figure 2(a1)	0.5720	0.6781	0.5722
Figure 2(b1)	0.9557	0.9231	0.8861
Figure 2(c1)	−0.0368	−0.0111	0.0135
Figure 2(a2)	0.8702	0.6628	0.6315
Figure 2(b2)	0.9045	0.9315	0.8633
Figure 2(c2)	−0.0351	0.0396	−0.0260
Figure 2(a3)	0.9939	0.9859	0.9791
Figure 2(b3)	0.9548	0.9565	0.9079
Figure 2(c3)	0.0004	−0.0121	0.0128

**Table 3:** The information entropy of original and cipher images.

Images	IE
Figure 2(a1)	7.1273
Figure 2(b1)	7.5693
Figure 2(c1)	7.7459
Figure 2(a2)	7.1208
Figure 2(b2)	6.7040
Figure 2(c2)	7.7289
Figure 2(a3)	7.4457
Figure 2(b3)	7.5046
Figure 2(c3)	7.7578

**Table 4:** The key space comparison results.

Encryption Algorithms	Key Space
Proposed algorithm	$2^{256\times 256+15}$
Reference [28]	$2^{298}$
Reference [29]	$2^{299}$
Reference [34]	$2^{375}$
Reference [35]	$10^{248}$
Reference [36]	$10^{58}$

**Table 5:** The computational complexity of each step and overall complexity.

Step 1	Step 2	Step 3	Step 4	Step 5	Overall Complexity
O(n)	O(n)	O(n)	$O\left(n^{2}\right)$	$O\left(n^{2}\right)$	$O\left(n^{2}\right)$
